# ‘Believe in me and I will believe in myself’, a rural Australian health service learns how to *mangan dunguludja ngatan* (build strong employment) for Aboriginal and Torres Strait Islander people: a qualitative study

**DOI:** 10.1186/s12960-019-0384-2

**Published:** 2019-06-19

**Authors:** C. A. Opie, B. Gibson-Thorpe, C. Lees, H. M. Haines

**Affiliations:** 1 Echuca Regional Health, Echuca, Australia; 2 University of Melbourne, Shepparton, Australia

**Keywords:** Employment, Aboriginal, Indigenous, Rural health service, Australia, Cultural inclusion

## Abstract

**Background:**

Australian Aboriginal people have higher rates of unemployment and poorer health than non-Aboriginal Australians. Historical segregation policies that spanned 60 years negatively impacted workforce inclusion. A Victorian regional health service recently developed an Aboriginal Employment Plan (AEP) targeted to reach 2% employment of Aboriginal people by 2020. This study aimed to identify strategies that will build strong Aboriginal employment.

**Methodology:**

A qualitative research protocol was designed. Purposive recruitment of people with a vested interest in the growth of Aboriginal employment at the health service participated in focus groups and individual interviews.

**Results:**

Twenty-four people including local Elders, past and present Aboriginal employees, key community stakeholders and health service executives participated. Learnings from the past, the present and strategies for the future emerged from two important stories: (1) the story of a strong group of local Aboriginal people who successfully approached the matron of the hospital in the early 1960s for employment. (2) The story of the ‘verandah babies’.

**Discussion:**

The history of the health service in question demonstrated the power of the possible with a self-determined group of Aboriginal people, who, in the face of cultural inequity, achieved employment at the health service. The opportunity for healing and a new start was illustrated by the story of women who gave birth on the verandahs due to their exclusion from the main hospital. Today, the ‘verandahs’ have been replaced with a modern hospital decorated with Aboriginal art, expressing cultural safety and inclusion, presenting fertile ground for strengthening and sustaining Aboriginal employment.

**Conclusion:**

Eleven strategies have emerged from three themes; safety, equity and pathway, offering mainstream health services insight into how to *mangan dunguludja ngatan* (build strong employment). Cultural safety can be achieved through acknowledging the past and reconciling that through engaging, partnering and collaborating with the Aboriginal community. Visual representations of culture and participation in celebratory activities engender awareness and understanding. The development of local, flexible career development pathways for Aboriginal people facilitates a ‘sense of belonging’ to the health service and a dual ‘sense of pride’ within the community: whereby the Aboriginal person feels proud to represent their community and the community is proud to be represented. Cultural equity is facilitated through mutual learning and reciprocal understanding of difference.

## Background

It is long understood that colonial legacies have caused intergenerational trauma that has eroded cultural spirit and identity among Australian Aboriginal and Torres Strait Islander people (herein respectfully referred to as Aboriginal people) [1], a persisting factor in contemporary socioeconomic disadvantage, discrimination and racism [2]. Some improvements in empowerment and control as a first nation race have been experienced in recent decades [3]. Yet, a 10-year national commitment to *Closing the Gap* indicates that Australians cannot sit idle [4]*.* According to the *2018 Prime Minister’s Closing the Gap Report*, Aboriginal literacy and numeracy, attendance at school, employment rates and life expectancy are not on track [4]. Nationally, over half of the Aboriginal population was unemployed compared to only 28.2% of non-Aboriginal people in 2016 [4]. Research has shown that education and income are inversely linked to morbidity and mortality rates [5, 6] and that efforts that enable an increased rate of employment among Aboriginal people can contribute to equitable health outcomes [7, 8].

In response to the disparity between Aboriginal and non-Aboriginal employment rates, the public sector in one Australian state (Victoria) developed *Karreeta Yirramboi* (Karreeta is a *Gunditjmara* word meaning ‘grow’ and Yirramboi is a *Taungurung* word meaning ‘tomorrow’), a 2010–2015 employment and career development action plan [9]. Action area 1 aims to build ‘… pathways between education and employment’ [9], action area 3 focuses on creating supportive culturally inclusive workplaces. In an Australian context, a culturally inclusive workplace focuses ‘… on the interplay between Aboriginal and non-Aboriginal cultures’ [9]. Cultural awareness is gained through recognising one’s own culture (self-beliefs, values and perceptions) and understanding how this influences how other cultures, not just Aboriginal, are interpreted and evaluated [9]. Cultural competence exists in a workplace that respects cultural difference and is effected through appropriate behaviours and communication strategies [9]. Aboriginal and non-Aboriginal staff benefit through learning ‘… cultural norms outside their own experiences and how these affect behaviours and expectations at work’ [9]*.*

Two further documents sit alongside *Karreeta Yirramboi*, namely the *Victorian Aboriginal Economic Strategy 2013–2020* [10] and the *Victorian Aboriginal Affairs Framework 2013–2018* [11] each with a focus upon education and income. *Karreeta Yirramboi* aimed to increase the Aboriginal representation in the Victorian public sector to 1% by 2015 [9]. The Victorian regional public health service described in this study developed an Aboriginal Employment Plan (AEP) targeted to reach 2% employment of Aboriginal people by 2020. This target was chosen for being greater than baseline (1%) and is closer to parity with the recorded Aboriginal population in the district (2.4%) [12]. However, this figure is still well below the 6% of Aboriginal people recorded as having accessed the health service emergency department or admitted as an in-patient over the past 10 years. Located approximately 90 km from the nearest tertiary health service, the studied sub-regional health service recently underwent a major redevelopment of the integrated acute hospital, now incorporating a sub-acute (in-patient rehabilitation) unit to complement the community palliative care, primary care and residential aged care service. A total of 134 beds, servicing an estimated catchment of 45 000 people resulted.

One Aboriginal Health Liaison Officer (AHLO) is employed at the mainstream health service. Two Aboriginal Community Controlled Health Organisations (ACCHOs) exist within the broader community, providing an array of primary and community healthcare. Aboriginal health workers provide a critical link between mainstream health services and the community, through provisions of culturally appropriate advocacy and comfort [13, 14]. For an Aboriginal person, ‘advocacy and comfort’ are key elements of ‘cultural safety’ and cultural safety enables improved adherence to healthcare advice [13, 14]. Employing Aboriginal people therefore has a dual effect; primarily improving the health and wellbeing of the individual employee through provision of an income [7, 8], as well as the health and wellbeing of the community at large through enhancing the cultural safety of the organisation [15, 16].

To date, at this regional health service, employees have not been routinely asked their Aboriginal status; hence, there is no evidence available to show rates or trends over time. Multiple employment vacancies are known to exist at all times at the health service; reportedly, however, few Aboriginal people apply. It is poorly understood why Aboriginal people refrain from applying for roles at the health service; hence, the aim of the study presented here was to learn how a mainstream health service can *mangan dunguludgja ngatan* (build strong employment).

## Method

### Study design

The study design was informed by the Australian Health Ministers’ Advisory Council (AHMAC): National Aboriginal and Torres Strait Islander Health Workforce Strategic Framework [8]. Health sectors are guided nationally to ‘work with local Aboriginal and Torres Strait Islander communities to co-design and co-deliver workforce programs and initiatives’ (p9 [8]) in an attempt ‘to provide culturally-safe and responsive workplace environments’ (p9 [8]) for Aboriginal people. BG-T is an Aboriginal woman and was employed at the health service as the AHLO at the time of the study. As the principal investigator, BG-T had an established relationship with the local Aboriginal community, engendering a sense of trust and safety in the research. Engaging directly with the Aboriginal community ensured that those being researched were actively involved and able to directly inform the strategies or actions required to build strong employment among their people, a process known as participatory action research [17].

Permission for the study on country was sought and granted by the local Elders. Advice for the study design was provided by Aboriginal members of the University of Melbourne (UoM) Aboriginal Research unit at the Department of Rural Health (DRH), given the University campus is on country. A qualitative study design using focus groups in the first instance was chosen as a means of group ‘yarning’ and gathering information directly from the people and stakeholders that are relevant to strengthening an Aboriginal workforce. Aboriginal people use the term ‘yarning’ to describe the process of oral communication that means to tell and share stories and information [18]. Yarning in a focus group or ‘collaborative yarning’ is a legitimate form of collecting data with Aboriginal people, given it enables a relaxed, informal and culturally safe space that allows ideas to bounce between participants as concepts build [18, 19]. Collaborative yarning allows the researcher and the participants to develop a respectful partnership in the knowledge creation [20]. The Malka Room was chosen as the venue for the focus group, further enabling cultural safety. *Malka* means ‘shield’ or ‘protection’ in the local Aboriginal language and was chosen by the community as the name for the Aboriginal meeting place built during the recent redevelopment of the health service. The Malka Room is imprinted with the local animal totem in the design of the ceiling, the walls and the furniture, engendering cultural safety. Telephone interviews were offered to participants unable to attend a focus group. Human experiences are often explored using qualitative methods as it offers a structural process to learn the inner life of people as it emerges in the dialogue, value free [21].

### Data collection

A purposive sampling method was used to engage four population groups as a first co-design step in exploring and collaborating with both stakeholders and end users [22, 23] to inform action-based strategies that strengthen the health service’s Aboriginal workforce:

1. Local Aboriginal Elders (may include previous employees)
2. Past or present Aboriginal employees
3. Key community stakeholders (workforce, education and training agencies and ACCHO representatives)
4. Regional health service executive officers

Participants were invited by the principal investigator to share stories in a focus group in the first instance, and if unavailable, a telephone interview was offered. Semi-structured questions were informed by the aforementioned *National Aboriginal Health Workforce Strategic Framework* [8] and developed by the principal researchers. Questions varied in accordance with participant group (Table 1.):

**Table 1:** Interview questions

Local Aboriginal Elders: 1. Describe what it was like working at the hospital? 2. Tell me about the type of work you did at the hospital? 3. Tell me what the health service might be able to do to encourage Aboriginal people to seek employment at the hospital? 4. Would you like to share your stories/memories and history of the hospital?	
Past and present employees: 1. Describe how you found out about your employment opportunity at the health service? 2. Talk about how you applied for the job? 3. Tell me about the interview process (if offered)? 4. For those of you who have or are still working at the health service, tell me about the experience? 5. Tell me how the health service may be able to make improvements (if any) for the future?	
Key community stakeholders: 1. If an Aboriginal person seeks your advice about careers, tell me whether you would suggest or discuss career options at the health service? 2. Tell me about your experiences in assisting an Aboriginal person through the application process at the health service? 3. Talk to me about what is already happening and working in this region with regards to Aboriginal employment in health?	
Health service executive officers: 1. How would you describe the health service’s current performance in relation to the recruitment and retention of Aboriginal and Torres Strait Islander people within the hospital? 2. Does the health service have specific targets/goals in mind for Aboriginal and Torres Strait Islander employment within the hospital (next twelve months, 5 years, 10 years)? 3. What systems and processes does the health service have in place to support Aboriginal and Torres Strait Islander employment (recruitment and selection, on-boarding, retention, etc.)? 4. What changes or improvements would you need to make to realise the targets/goals identified in question 2?	

In accordance with the National Statement on Ethical Conduct in Human Research [24], it was deemed imperative by the presiding ethics committee that BG-T refrain from data collection due to a conflict of interest, given her Aboriginal status and employment at the health service. An independent consultant with a longstanding and respected history working with local ACCHOs was consequently recruited to conduct the focus groups and telephone interviews, adding rigour to the study design [25].

### Recruitment

An overview of the study aim and methodology was presented to each of the target population groups by the principal investigator, in person, inviting their participation in a focus group or an individual telephone interview. Participation in the study was voluntary and explained in the Plain Language Statement (PLS), as provided to all participants. Elder permission was sought and granted to print the local Aboriginal animal totem as a background image on the PLS to express cultural safety. Interested participants were encouraged to contact the principal investigator. Understanding of and consent to participate in the study was recorded in written format at the time of participation.

### Data analysis

Stories voiced in focus groups 1 to 3 were written on paper or a ‘storyboard’ in full view of participants for data transparency and member checking. Shay [19] reports that ‘storyboards’ are an ideal methodology when conducting focus groups with Aboriginal people, enabling cross-checking and co-analysis at the time of data recording. It is felt that visually representing the data in real time improves engagement and provides an opportunity for corrections more readily than the traditional audio recording and transcription review process [19]. At the end of each focus group, the stories were summarised and checked for clarity of meaning with all participants. No corrections were required. Focus group 4 was facilitated via telephone conference, whereby all research participants were together face to face, including two of the principal investigators (CO and BG-T) while the independent consultant moderated the discussion via telephone while taking notes. Field notes, a useful method of describing and interpreting data [26], were recorded during all focus groups by a member of the research team (CO) to capture the expressions and interactions among the groups, in particular some valuable quotations voiced.

The consultant collated the storyboard and interview data and reported findings to the research team. All data (including field notes) was then organised chronologically by CO and BG-T (past, present, future) and conceptualised for a general pattern in cultural meaning, a form of narrative analysis [27]. Data was then coded and summarised into broad themes, enabling readers to contextualise strategies for strengthening Aboriginal employment in a healthcare setting.

## Results

Twenty-four people participated in focus groups and/or telephone interviews (Table 2). Twelve participants identified as Aboriginal.

**Table 2:** Focus group and telephone interview participants

Method	Target population	Participants (*N* = 24)	Aboriginal people (*n* (%))
Focus group (1)	Local Aboriginal Elders (note: all 3 Elders are also a past employee)	3	3 (100%)
Focus group (2)	Past or present Aboriginal employees	5	5 (100%)
Focus group (3)	Key community stakeholders	6	1 (16.7%)
Focus group (4)	Health service executive officers	7	0 (0%)
Telephone interview	Local Aboriginal Elder (1) (note: also a past employee) Key community stakeholder (1) Past or present employee (1)	3	3 (100%)

Stories gathered from all participants were categorised into three major learnings relating to Aboriginal employment at the health service: past, present and future.

### Learnings from the past

Cultural inequality was identified by Aboriginal participants as a persistent story among the contemporary Aboriginal community, having experienced firsthand the 60 years Commonwealth Segregation Policy [3]. The term ‘verandah baby’ created a significant level of yarning among participating Elders, ‘I was a verandah baby; my younger sister was also …’ [P7—Aboriginal], indicating that segregation is well imprinted in the memories of the local Aboriginal community. One Aboriginal Elder recalled being quite young for her first delivery and did not question being segregated to the outside of the health service until her second birthing experience: ‘my eldest boy, he was a verandah baby, being young I never took notice, but when I had my [second baby], I was in the [designated] Room’ [P8—Aboriginal]. Once the verandahs were no longer used, Aboriginal mothers continued to be segregated into a designated room within the building.

Unwell Aboriginal children were also segregated onto the verandah before being segregated indoors, recalled as a frightening experience for one participant*:* ‘I wasn’t in the children’s ward with the other children, I was shitting [sic] myself at night time … … I was out there by myself [verandah]! [However] I knew I was on my ancestors land and so I was safe’ [P13—Aboriginal]. Traditional cultural practices and beliefs provided some comfort and an ability to soften the reported heartache that segregation triggered.

Over time, Aboriginal people did notice and question segregation, stating that the inequality created a barrier between the Aboriginal community and the health service, resulting in a level of suspicion that impacted trust and cultural safety. Aboriginal people began to disengage ‘I want equal rights to health, [segregation] made [the] next generation question. I heard a bloke say ‘I won’t go to those institutions, “cos they never let my mother in”’ [P13—Aboriginal]. Passive resistance and distrust was later met with a growing movement toward self-determination, reported by participants as being in the 1960s, when community members approached the Matron of the health service, to ask for employment. ‘We said to her [Matron], “accept us … … we live in town and we are responsible people”’ [P5—Aboriginal]. Aboriginal people from the local missionary community were keen to overcome the division that the Segregation Policy had caused between Aboriginal people and the health service: ‘there were six of us working there in the end... … being employed at the hospital broke down the “us and them”’ [P4—Aboriginal].

The physical walls that had segregated Aboriginal people at the health service were splintered through their presence as employees, as one Elder remembers: ‘having people from [mission] working at the hospital meant that the community would go there’ [P4—Aboriginal]. Trust was being rebuilt between non-Aboriginal health professionals and the community. With trust came cultural safety and Aboriginal people began to accept an admission ‘within’ the hospital, rather than external to it: ‘even the doctors, some of them helped our people to come into the hospital’ [P13—Aboriginal].

Employing Aboriginal people signified bilateral rewards for the community; on the one hand, the Aboriginal presence meant ‘cultural safety’ and ‘healing’ while enabling an opportunity for the employees to undertake their ‘cultural duty’. Aboriginal employees were the face of their community, and regardless of the job they were employed to do at the health service, for example cleaning or cooking, they dedicated their breaks to visiting Aboriginal patients to supplement their care: ‘in the old days, we would visit the Elders and do chores for them in our breaks’ [P5—Aboriginal]. Undertaking ‘chores’ relieved the pressures on the families of Aboriginal patients who may have had limited access to the health service. Aboriginal employees were identified as role models who were proud of their cultural duty as ‘carers’. They were revered by their broader community and identified as facilitators of healing for an Aboriginal in-patient.

They [Aboriginal employees] took pride in the role they had in the hospital—that had a good impression on me and how I took [understood] your role as a community member. Mum [Aboriginal employee] stayed with our people so they would stay, ‘cos our people didn’t want to stay. They [Aboriginal patients] never had anybody to go to … … these folks [Aboriginal employees] did those jobs … … they were domestics but they [health service] never had Hospital Liaison, so they did those jobs and extra [work duties] for our people. Even Aunty [Aboriginal employee] who worked in the kitchen. Just to see those faces, your healing is not just the pill, but it’s seeing those faces and just knowing that and coming in gives you comfort.

[P7—Aboriginal].

### Learnings from the present

Executive officers agreed that a longstanding commitment (10 years) to developing a culturally inclusive workplace for Aboriginal people has been evident in the health service strategic plan. It was felt, however, that efforts take time to see tangible results: ‘we [health service] are doing significantly better now than we were 5 years ago’ [P18—Non-Aboriginal]. One significant achievement voiced was having an Aboriginal woman on the health service Board: ‘we [health service] have been very fortunate that we have had a very well-known Aboriginal lady on our Board for many years and in fact until very recently [she] was the Chair of our Board’ [P21—Non-Aboriginal]. It was reported by an executive officer that current successes create fertile ground for potential: ‘we [health service] haven’t yet recruited an Aboriginal doctor, which is an achievement we would certainly aspire to in the future … … but I think the environment that is being referred to by my colleagues … … is conducive to future success, in that important area of having an Aboriginal doctor in this community … … which would be of great value to this health service and to the broader community’ [P19—Non-Aboriginal].

For the Aboriginal community, a strong example of a move toward social inclusion was demonstrated through the 2012–2015 physical redevelopment of the health service. The redevelopment was a turning point, an opportunity to create an environment that was culturally safe from a visual perspective. Local Aboriginal community members were engaged at the outset: ‘the Art Acquisition Advisory Group gave people the opportunity to look at the history and how to move forward by yarning about the style of the artwork and the new storyline that they wanted to share to heal the past’ [P6—Aboriginal]. Incorporating Aboriginal artwork into the new facility was reported by an executive officer as a complementary suite of visual strategies. For example, ‘Acknowledgement of Country’ plaques were designed by the local Aboriginal community and placed at each entry to the health service. All meetings were opened with an ‘acknowledgement of country’ as a sign of cultural respect. Finally, a designated ‘safe place’ [P6—Aboriginal] was built during the redevelopment of the health service and named the ‘Malka Room’ [P6—Aboriginal] by local Aboriginal Elders to represent a ‘shield of protection’ [P7—Aboriginal].

Another milestone included the development of the AEP and the Memorandum of Understanding (MoU) with two local ACCHOs: ‘our [health service] has a 2% Aboriginal Employment Plan by 2020 … … we also have some Memorandums of Understanding with local Aboriginal organisations and they are well aware of our target and we work together to try and reach that target’ [P22—Non-Aboriginal]. It was noted that historically the health service has not recorded the Aboriginal status of employees within standard recruitment processes, indicating that trends over time are not known. Anecdotally however, it is believed that there is a shift toward improving the numbers of Aboriginal employees: ‘we’re [health service] seeing increased numbers of Aboriginal people applying for general employment positions … … not just Aboriginal specific’ [P21—Non-Aboriginal].

Executive officers (*n* = 7) celebrated the AEP as a means of maintaining focus and an organisational wide commitment to increasing Aboriginal employment. One key strategy underpinning the focus was through employing a dedicated HR staff member, to seek funds to recruit and develop career pathways for Aboriginal trainees: ‘we’re recruiting our fourth [Aboriginal] trainee as we speak’ [P18—Non-Aboriginal]. A key role of the HR staff member was to partner with key stakeholders including employment agencies and ACCHOs. Building a strong community network enabled an ability to seek advice and respond to culturally sensitive issues as they arose, particularly in the beginning when multiple assumptions were made about employment expectations: ‘I think one of the really big learnings is that we made assumptions around Aboriginal community members that we had employed that they came from the same sort of supportive background as most of our mainstream employees’ [P20—Non-Aboriginal].

Employing Aboriginal trainees introduced challenges that differed to non-Aboriginal trainees: ‘we made assumptions about - everybody knows that you have to turn up on time, in your uniform and if you don’t or can’t present to work, you have to phone in and notify people, we took that as a given - until people didn’t turn up and we didn’t hear from them’ [P20—Non-Aboriginal]. The designated HR person was reported as an important link in determining the challenges through regular meetings with each trainee. Without talking to the Aboriginal trainees individually, it was stated that they would not have been able to learn how to ‘…support them to be successful in the role’ [P20—Non-Aboriginal]. Learnings facilitated a reflection on the assumption that traineeships are often provided to people who come from ‘…a solid home background and parental support’ [P20—Non-Aboriginal] that enables a sufficient safety net for trainees receiving a small wage. That safety net was found to be insufficient for Aboriginal trainees, ‘…we need to ensure we provide appropriate support’ [P20—Non-Aboriginal]. As an example, the health service purchased a bicycle for one Aboriginal trainee, enabling transport to work. Perceived mistakes were later realised as cultural learning: ‘[it was] easy to get discouraged going down this path—all too hard, [but then] when you get into it and hear the stories of these young people and their level of disadvantage … … you think “gosh the rewards outweigh the challenges”’ [P20—Non-Aboriginal].

Through regularly meeting with the Aboriginal trainees, an executive officer reported that they realised that once the initial ‘work ready’ [P23—Non-Aboriginal] challenges were overcome, new challenges existed. For example the ‘language spoken’: Aboriginal trainees indicated that mainstream health service employees ‘…speak a very formal language … … which other people find very difficult to understand’ [P20—Non-Aboriginal]. More than that, Aboriginal trainees disclosed that they needed someone to go to be able to discuss how difficult their day was or that they did not know what people were talking about. The Aboriginal trainees declared that it was very difficult for them to discuss matters with their non-Aboriginal managers, reporting that it was very embarrassing ‘…they felt they lost face … … they couldn’t acknowledge … … they couldn’t identify it, they were failing and they weren’t talking to us’ [P20—Non-Aboriginal]. Openly discussing the challenges directly with the Aboriginal trainees highlighted areas that needed strengthening: ‘…we identified very early that we needed to have some Aboriginal mentor support’ [P20—Non-Aboriginal].

The designated Aboriginal HR person and the AHLO were subsequently tasked with adopting a greater role in supporting the cultural needs of the Aboriginal trainees: ‘key people in HR and AHLO to go to allows an ability to pick up issues early and try to address them … … helps to overcome assumptions’ [P20—Non-Aboriginal]. Learnings however had to be mutual in character; Aboriginal trainees were encouraged to be upfront with their challenges by talking to their manager, the AHLO or the HR person who had to listen and respond sensitively. An executive officer reported that it was important to be ‘upfront’ with the Aboriginal trainees and be really ‘clear about what the avenues are, that this is not a shame thing, this is something that we can help with. We expect you [Aboriginal trainee] to have difficulties, we expect it to sometimes not go smoothly and that’s okay’ [P20—Non-Aboriginal]. One criticism of having provided a broad culturally safe network was reported as developing a new issue: ‘at times there has been confusion with the support [HR or AHLO] and the line manager’ [P21—Non-Aboriginal].

To combat confusion for Aboriginal trainees, executive members (*n* = 5) decided to broaden the safety net, through ensuring that all health service staff undertake cultural awareness training: ‘we [health service] are one of few organisations … … to have cultural training as mandatory … … for every single staff member’ [P24—Non-Aboriginal]. The health service reported introducing an online training package entitled, ‘Share Our Pride’ as developed by Reconciliation Australia [28]. Training specifically targeted areas where the Aboriginal trainees were employed, and importantly, those staff ‘…are continually offered opportunities to train further and learn more about Aboriginal culture’ [P22—Non-Aboriginal]. It was reported that training staff in cultural awareness has had a positive impact on the retention of Aboriginal trainees because non-Aboriginal managers are ‘more conscious of how they work with their Aboriginal team members’ [P22—Non-Aboriginal].

In parallel to the strategies employed to recruit and retain Aboriginal trainees, the health service Education Centre developed a greater focus on relationship building with the local secondary schools, exemplified with an annual Aboriginal Career Expo. The Expo has led to improved relationships with the community: ‘I had a father phone me … … somebody at the school talked to him [about Aboriginal traineeship] about how this might be a good pathway for his daughter’ [P20—Non-Aboriginal]. Promoting career pathways to secondary school students was supplemented with offering career progression such as a pipeline or a pathway to further education to current Aboriginal trainees. ‘We had one Assistant in Nursing who said ‘I always wanted to be a midwife’, so we aimed to build her role around her aspirations and the needs of the organisation … … this led to an increased flexibility in training and changing … … given the needs change as they [Aboriginal trainee] learn’ [P20—Non-Aboriginal]. Having trainees enthusiastic about being able to grow their skills and knowledge was reported by the executive group as a motivation to partner with training organisations, such as the [regional] Institute. Tailoring opportunities to ensure pathways are possible was reported as positive for Aboriginal employees: ‘give [Aboriginal] people a ‘vision’ of where they are going’ [P6—Aboriginal].

A final key strategy reported by the executive group (*n* = 4) as having led to present successes is the development of strong relationships with the Aboriginal community and an ongoing commitment to strengthening and growing the partnerships: ‘we’re [health service] involved in a lot of community committees … … we certainly ensure wherever possible that representatives from the local ACCHOs in particular are on those groups … … Aboriginal issues are always a part of our work’ [P21—Non-Aboriginal]. A key stakeholder and an executive member state that an added advantage of collaborative partnerships is the ability to develop the Aboriginal workforce together, through seeking opportunities for dual positions within the health service and ACCHOs: ‘we’re [health service] always thinking of how we can connect with local Aboriginal organisations when we have specific roles that might be available or placement opportunities or chances just to get together and share different training’ [P22—Non-Aboriginal].

### Learnings for the future

All study participants were united in acknowledging that the health service has indeed exemplified a contemporary commitment to cultural inclusion. It was reported, however, that continued vigilance is vital to ensure the health service is broadly known within the Aboriginal community as a culturally safe place to work: ‘although they [health service] have come a long way, it is still hard for Aboriginal people to get a job there’ [P5—Aboriginal]. Key stakeholders reported that the health service would benefit from advertising available positions in the places known to be readily accessible to the Aboriginal community: for example, in appropriate print media: ‘promote jobs in the [Aboriginal] mail and all the CoOps and write that “Aboriginal people are encouraged to apply”’ [P5—Aboriginal], or in waiting rooms: ‘jobs could be advertised in waiting rooms at [the health service], this may overcome barriers to no internet’ [P2—Non-Aboriginal].

Promoting the health service as a culturally safe workplace was a factor that two key stakeholders stated as being vital in attracting Aboriginal people as employees. It was reported that using existing Aboriginal staff as role models or ‘poster people’ at the Aboriginal Career Expo or displayed in public places within the health service, would support the traditional ‘cultural duty’ aspect that was historically recognised as powerful. A plaque identifying all past employees, in particular AHLOs, was also suggested.

In parallel to identifying methods to promote the health service as a culturally safe place to work, a current Aboriginal employee and key stakeholders agreed that an ‘Expression of Interest’ (EOI) may be a valuable alternative to a ‘job application’ as a primary means of engagement with the health service. Skills in writing a full job application and subsequent participation in an interview were reported as particularly challenging for Aboriginal people: ‘I can’t talk about myself [in an interview]’ [P17—Aboriginal]. A number of cultural safety factors were explored by the key stakeholders, each with a valued contribution. Interviewing Aboriginal people was stated as being most culturally appropriate if conducted ‘in a circle’ [P16—Non-Aboriginal], allowing an informal approach. One suggestion was to allow Aboriginal people to bring an advocate to an interview. It was also thought that having an Aboriginal person on the interview panel would be valuable and should be mandatory.

Local training and workforce agencies reported that Aboriginal employment could be strengthened if health services worked more collaboratively. For example, Aboriginal people seeking employment have significant existing support available through local workforce readiness providers, including school transition to the workplace programs, résumé and key selection criteria writing. Future workforce planning could be improved through collaboration between the health service and the local training and workforce agencies. A key stakeholder stated that better planning would allow improved skill development and work readiness: ‘if we [workforce agency] knew what positions were coming up, we could skill Aboriginal people accordingly’ [P12—Non-Aboriginal]. It was acknowledged that conducting a skills shortage analysis could prevent inappropriate employment of Aboriginal people: ‘just filling a position with an Aboriginal person is not the answer, if they don’t have the skill set or the credentials then they are being set up to fail’ [P14—Aboriginal]. Skill matching was considered vital by a current Aboriginal employee, to ensure the health service is matching the community demand or needs with the job: ‘[create] real and good jobs, not tokenistic jobs that are too big, like the AHLO role’ [P9—Aboriginal].

It was suggested by a key stakeholder that skill matching could be promoted with Year 8 and 9 students in local secondary schools, to allow for appropriate subject selection and potentially foster school retention. Four key stakeholders agreed that growing an Aboriginal workforce is a long-term vision with no immediate outcomes. One stakeholder stated that the Aboriginal Career Expo and the hosting of Aboriginal Allied Health Certificate III students (from local secondary college) have been positive. The stakeholder stated that targeted career events allow an ability to ‘touch and feel’ equipment and explore pathways. Further opportunities could be explored if Aboriginal students were able to ‘shadow’ a health professional for a day, stating that ‘Aboriginal people don’t get an opportunity to learn what they are good at’ [P11—Non-Aboriginal]. A current Aboriginal employee agreed: ‘[provide an opportunity] to try it [job] out’ [P17—Aboriginal].

A current Aboriginal employee reported that health services would also benefit from including ACCHOs in the process of analysing demand and ensuring that jobs are culturally appropriate. Particular reference was made to a felt lack of appreciation of gender-specific needs: ‘it comes back to management’s commitment to ‘men’s business’ and ‘women’s business’ and acknowledging the difference’ [P6—Aboriginal]. In Aboriginal culture, reproductive and sexual health is traditionally discussed with an Elder of the same gender [29]. Aiding the cultural learning therefore through strengthening relationships with local ACCHOs was reported as offering a perceived missing element in the mainstream health service current employment processes. ‘Sorry business’ was exemplified as an area of cultural sensitivity that health service executive and managers could learn more about. Sorry business refers to a period of ‘cultural mourning’ following the death of a family member among Aboriginal people [30]. Out of respect, families are to be left alone throughout mourning [30]. It was felt that when a mainstream organisation does not respect sorry business and afford leave accordingly, then Aboriginal staff will resign because the mourning period is a cultural duty that takes precedence.

Sorry Business – depends on a person’s standing in the family … … their responsibility that different ones take on ‘cos it’s expected by the family ‘cos of their role in the family – that needs to be taken into consideration. Being flexible. ‘Cultural Business’ is allocated in ACCHO on top of sick leave and annual leave – more sympathetic [than mainstream]. Offer ‘Cultural Business’, this will retain and keep good staff … … put it in the job advert. [P7—Aboriginal].

Establishing opportunities to build the relationship between the health service and ACCHOs was considered readily achieved through regular visits: ‘come out for a chat, just come out for a chat’ [P13—Aboriginal]. It was stated that Aboriginal employees would benefit from developing networks with Aboriginal staff at local ACCHOs as it allowed a sense of ‘connection’ to their community and mitigates isolation: ‘there are ups and downs working in mainstream, you yearn for your Aboriginal peers’ [P6—Aboriginal]. In parallel, current Aboriginal employees stated that the health service would benefit from enabling existing Aboriginal employees to lean on one another: ‘an Aboriginal Staff Network needs to be established’ [P6—Aboriginal]. Another Aboriginal employee added that ‘group support works best – need to “vent” if something is pissing [sic] you off’ [P17—Aboriginal]. An Aboriginal Staff Network was suggested as a means of providing Aboriginal employees with informal support, mentoring and potential opportunity for planning social activities to develop connectedness.

Improvements in cultural learning would facilitate an understanding of cultural difference. An Aboriginal stakeholder stated that it was important to understand that Aboriginal people are ‘story tellers’ and learn by being told and shown rather than by written instruction: ‘they’re [Aboriginal people] better with hands on—the theory comes last’ [P13—Aboriginal]. A current Aboriginal employee agreed, ‘if you demonstrate, I’ll pick it up first go, but if you give me a piece of paper, yeah … … I won’t’ [P3—Aboriginal]. An Aboriginal stakeholder added that Aboriginal people need to have a clear understanding of the job expectations, including specification of start/finish times and breaks, rather than assume that this is known. Critical to success in sustaining an Aboriginal workforce is to show that the organisation is ‘not giving up on them [Aboriginal Employee] … … “believe in me and I will believe in myself”’ [P7—Aboriginal].

Key stakeholders encouraged the health service to learn from other Australian businesses that have had success in growing an Aboriginal workforce; both Telstra Corporation Limited (a telecommunications company) and the Woolworths Group Limited (a major grocery store chain) were cited. It was reported that Woolworths Group Limited provide management staff with a 3-day course in cultural awareness, allowing greater confidence, trust and respect in the Aboriginal employee’s relationship with their manager.

Aboriginal stakeholders were keen to see the health service invest more in Aboriginal cultural events such as National Aborigines and Islanders Day Observance Committee (NAIDOC) celebrations, particularly events occurring in the local National Park each year. It was further stated that the local Aboriginal community would hold the health service in greater esteem if a Reconciliation Action Plan (RAP) was developed and publically announced to show an ongoing commitment to inclusion. Actions outlined in the RAP could be promoted at community events, including NAIDOC celebrations.

An additional sign of cultural respect could be achieved by including the Aboriginal history on the health service timeline that has been developed and displayed in the new health service facility foyer. A current Aboriginal employee stated that through publicising the history and including the days of segregation, the health service would be acknowledging the past and enabling Aboriginal people to feel that the future will be different.

Learnings from the past, present and future were summarised into three major themes by the authors with accompanying strategies that offer insight into how a mainstream health services can strengthen an Aboriginal workforce (Table 3).

**Table 3:** Summary of themes and strategies to *mangan dunguludja ngatan* (build strong employment)

Theme	Descriptor	Selected quotations	Strategies
Safety	Acknowledgement of the past and reconciling for the future:		
	• Cultural inclusion and healing • Physical (demolish and rebuild) • Visual trust • Participation—in Aboriginal celebrations • Engagement—visit Aboriginal services • Community connection sustained within and external to the workplace	‘…just to see those faces, your healing is not just the pill, but it’s seeing those faces and just knowing that and coming in gives you comfort’. [P7—Aboriginal]. ‘There are ups and downs working in mainstream, you yearn for your Aboriginal peers’ [P6—Aboriginal].	1. Embed a commitment to strengthen the Aboriginal workforce to facilitate healing within the building and external to it 2. Co-design buildings and physical spaces with the Aboriginal community to ensure cultural safety from a visual perspective 3. Participate in Aboriginal celebrations as a sign of respect and reconciliation 4. Regularly engage with the Aboriginal community within their organisations to learn the culture 5. Embed systems of support for Aboriginal employees to ensure social inclusion and cultural connectedness both within and external to the organisation, e.g. an Aboriginal Staff Network
Pathway	Acknowledgement of opportunity:		
	• Motivation • Flexibility • Sense of belonging • Value (cultural role models)	‘We had one Assistant in Nursing who said “I always wanted to be a midwife”, so we aimed to build her role around her aspirations and the needs of the organisation … … this led to an increased flexibility in training and changing … … given the needs change as they [Aboriginal trainee] learn’ [P20—Non-Aboriginal].	6. Develop flexible goal directed opportunities for skill development with potential and existing Aboriginal employees 7. Promote the successes/value of Aboriginal employees to the broader community
Equity	Acknowledgement of difference (mutual learning):		
	• Cultural needs/learning: seeking, applying and retaining employment; sorry business • Clear expectations/learning differences • Cultural training • Relationships • Trust-partner with Aboriginal community (MoU) • Interagency workforce planning; gender needs (men’s business/women’s business)	‘…believe in me and I will believe in myself’ [P7—Aboriginal]. ‘...we had to look at their [Aboriginal employee] personal situation and make sure that they had someone cultural to go to [while at work]’ [P20—Non-Aboriginal].	8. Co-design recruitment processes with the Aboriginal community to ensure cultural safety 9. Embed cultural training for non-Aboriginal employees 10. Develop meaningful (outcomes focused) relationships with the Aboriginal community to enable trust 11. Collaborate with the broader community to identify opportunities to develop the Aboriginal workforce together

## Discussion

Yarning with twelve Aboriginal and twelve non-Aboriginal stakeholders interested in the sustainable employment of Aboriginal people at a regional Australian health service, has provided significant insight into how past, present and future learnings may help to strengthen Aboriginal employment. Learnings led to the development of three significant cultural themes: safety, pathway and equity with each hereby explored in turn.

### Safety

Past learnings indicated that cultural inequality as it existed throughout much of the twentieth century in Australia as a result of the Segregation Policy (p22 [3]) was felt by local Aboriginal people who remember birthing their babies on the verandah of the hospital [31]. Segregation meant that healthcare was delivered on the outside of the health service for Aboriginal people who at that time found solace in their cultural beliefs and their relationship with their ancestral land. The connection with the land facilitated healing, in spite of the physical divide. Over time, however, Aboriginal people grew suspicious of mainstream health services, a suspicion that negatively impacted a sense of trust, safety and access. More than a physical divide resulted, rather a gap in health care provision between Aboriginal and non-Aboriginal people. Findings indicate that well before modern policy directives, the first step toward culturally healing past inequalities at this local health service was initiated by the very people who were negatively affected by it.

Akin to the growing movement toward ‘self-determination’ that later became nationally legislated in 1972–1975 (p22 [3]), local community members adopted a direct grassroots approach that saw a number of Aboriginal people being employed at the health service during the 1960s. Upon reflection, it was clear to the local Aboriginal community that this powerful step enabled, firstly, a positive shift in consciousness toward historical acceptance and, secondly, the building of trust and safety for Aboriginal people to begin physically healing *within* the building, rather than external to it.

According to Dockery [32], both educational attainment and employment among Aboriginal people are positively associated with strong cultural attachment. Findings presented here suggest that the local Aboriginal women, who exemplified self-determination and successfully sought employment at the health service decades ago, during an era of significant cultural inequality, were women who had overcome the suspicion. It may be implied therefore that they felt safe and well attached to their culture. Employed Aboriginal people at the health service at that time were strong role models for their community and understandably, began to break down the divide between ‘us and them’ [P4—Aboriginal]. Elders remember that concerted efforts led to six Aboriginal people being employed at the hospital. Today, it is unknown how many Aboriginal people work at the health service, given the on boarding processes do not capture Aboriginal status. Without recording Aboriginal status, attempts to strengthen the Aboriginal workplace cannot be measured.

Kareeta Yirramboi launched a concerted effort into developing economic growth among Aboriginal people [33], enabling the health service to review their local relationship with the Aboriginal community. Strength was evidenced by the longstanding membership of an Aboriginal person on the Board (and Chairperson for much of the current tenure). According to the first domain of the *Cultural Respect Framework*, as developed by AHMAC, Aboriginal inclusion in governance reflects a systemic organisational approach to cultural safety and responsiveness [34]. Three additional health service milestones reflected organisational leadership, namely, the cultural engagement during the physical redevelopment of the site, the signing of a MOU with each local ACCHO and the introduction of the AEP.

The health service was uniquely placed during the redesign and rebuild of the facility, to strengthen existing relationships with the Aboriginal community. Physically demolishing the very building that retained the ‘verandahs’ presented a symbolic opportunity to make amends for past wrong doing. An Art Acquisition Advisory Group was engaged to support the appropriate use of artwork, the installation of *Acknowledgement of Country* plaques at all entrances and the development of the *Malka Room*, a safe place for Aboriginal people. Foley [35] reports that prominently placed Aboriginal visual statements provide a philosophical, political and intellectual foundation pertinent to building inclusive pride, thus representing a commendable step forward in developing inclusion. Using artwork and displaying both the Aboriginal and Torres Strait Islander flags are both examples of communicating a culturally safe environment to the community—domain 2 in the *Cultural Respect Framework* [34]. Participating in Aboriginal-specific cultural celebrations, for example NAIDOC week, was also considered a step toward reconciling the past by the Aboriginal stakeholders in this study. The Northern Territory (NT) Primary Health Network (PHN) state in their Reconciliation Action Plan (RAP) that all Aboriginal and non-Aboriginal employees ought to be provided with opportunities to participate in NAIDOC activities as a fundamental strategy in gaining local cultural knowledge and understanding [36].

### Pathway

Preliminary attempts to *mangan dungaludja ngatan* (build strong employment) at the health service and achieve 2% employment of Aboriginal people by 2020, included multiple strategies aimed at Aboriginal youth. Aboriginal career expos, traineeships that incorporate a pipeline or pathway into higher education close to home, were described in this study by health service executive officers as major achievements. Certainly, building the capacity of Aboriginal employees and offering career progression is recognised as an organisational commitment to growing and retaining Aboriginal people [34] thus exemplifying a dedication to the AEP.

A regional Queensland health service identified in their 2016–2018 Cultural Capability Plan that fostering relationships with training providers and nurturing employment pathways for Aboriginal people is a key priority in building a dependable and sustainable healthcare system [37]. Carson and McConnell [38] found in their examination of Census data relating to remote NT Aboriginal health and community service employment that workforce growth can be sustained if Aboriginal people are engaged in educational opportunities pertaining to professional practice *within* their community, more so than accessing education *outside* of their community. It was also found in this study that when training providers are close to the home of an Aboriginal person, enhanced engagement results.

Mainstream health services were encouraged in this study to promote the successes of employees to the broader community as a means of providing an Aboriginal employee with a ‘sense of belonging’ to the organisation. Promoting Aboriginal employees during career expos as an example builds on the earlier concept of ‘role modelling’ and the sense of ‘cultural duty’ that the Elders discussed. In addition, it engenders self-pride in the Aboriginal employee and a belief that the organisation trusts and understands them ‘…believe in me and I will believe in myself’. The Heart Foundation in Western Australia (WA) found that both trust and flexibility are essential ingredients in supporting the personal development of an Aboriginal employee in a mainstream workplace [39].

### Equity

It was clear from present findings that supporting Aboriginal young people in their career development requires consideration of their home life and provision of support unique to this population group. Maru and Davies [40] explain that significant cultural factors that bind an Aboriginal person with family and kin networks can act as major facilitators and/or inhibitors relating to Aboriginal employment. Stories heard through the Roche et al. [41] study with Aboriginal alcohol and other drug (AOD) workers across Australia agree that often personal lives are indeed complex. Most workers reported being ‘…single parents or responsible for dependent children, elderly and extended family members. Many had experienced significant bereavements, domestic violence and personal or family problems with alcohol or drugs. The high prevalence of premature deaths and suicides in communities resulted in profound loss and grief... From an Indigenous perspective, loss and grief encapsulates ancestral, intergenerational, suppressed and unresolved grief often not well understood by mainstream colleagues’ [41].

Although the complexities of the personal lives of an Aboriginal person may be an important consideration for mainstream employers, it is worth noting that the Aboriginal employees studied by Roche et al. believed that there are strengths in having strong kinship networks, as it allows a deeper understanding into cultural issues, empowering worker responsiveness to community need [41]. Similarly in this study, mutual learning regarding cultural difference led to a realisation that true engagement with Aboriginal employees can lead to rewards that outweigh the challenges. It is a learning that is reciprocated, revisited regularly and ongoing to ensure equity in the workplace. Aboriginal AOD employees declared that it was vital to job satisfaction that they felt a strong level of support in their workplace, where peers and managers are culturally aware [41, 42]. Aboriginal health workers employed in a mainstream health service experience little separation from their community and their workplace duty to continuously advocate and create cultural safety, ultimately increasing the risk of emotional exhaustion and burnout [41, 42]. Culturally strong and flexible workplaces enable early identification of burnout and the need for additional unexpected support such as time off for ‘sorry business’ [39, 41, 42]. In Australia, compassionate leave entitles any full- or part-time employee to 2 days paid leave [43]. Ceremonial leave (‘sorry business’ entitlement) is often additionally considered in awards afforded to Aboriginal people [43] where up to 10 days unpaid leave may be available each year [44].

Over time, the mutual learnings about cultural difference were found to enable the employer in this study with an ability to have reciprocal conversations with Aboriginal employees, where clear expectations are discussed. Watson et al [45] also found in their Queensland study with Aboriginal child health workers (CHW) that performance was considered better if clear roles and responsibilities were outlined, learning was supported and suitable resources were made available. Peer mentoring was discovered by Browne and colleagues [46] in their evaluation of a mentoring relationship between Aboriginal health workers (AHWs) and allied health assistants (AHAs) as a valuable means to improving practice through two-way learning and importantly reciprocity in developing cultural competence. Reciprocal cultural learning in itself provides justification and value for collaborating with local Aboriginal organisations, conducting workforce planning together and seeking dual positions between the health service and local agencies, a suggestion made by key stakeholders in this study.

Developing a partnership or a MoU with an ACCHO is one step toward developing meaningful relationships that benefit mutual learning. It was equally important to the Aboriginal employees in this study that they too develop meaningful relationships with their community through both local agencies (outside their organisation) and each other within the health service. Mainstream employment was reported as being isolating when only few Aboriginal people are employed, indicating that an informal Aboriginal Staff Network would bridge that gap by ensuring interaction. Wood et al. [39] agreed that although most employees require nurturing and support to improve performance, the need is magnified among Aboriginal employees, particularly those working in mainstream organisations. Both *Karreeta Yirramboi* and the national strategic framework for developing the Aboriginal health workforce indicate that mentoring support and Aboriginal networks are important for retaining Aboriginal employees by facilitating career progression and knowledge development [8, 33]. However, little attention has been paid to having a network simply for connection with one another to mitigate isolation in mainstream workplaces, an outcome that warrants acknowledgement. Certainly, the health service has taken a strong leadership stance with embedding mandatory Cultural Awareness Training (CAT), aligning with the third domain: ‘Workforce Development and Training’ of the *Cultural Respect Framework* [34]. Importantly, however, Westwood and Westwood [47] found in their evaluation of either half or two full days of CAT programs offered in New South Wales (NSW), that CAT is often too broad and, at times, inadequately overcomes any predisposing attitudes or stereotypes toward Aboriginal people. Authors did however emphasise that embedding CAT for all staff, with an extended version of training in cultural competence offered to key staff, particularly managers, where broader leadership and accountability in improved health outcomes are expected, may be beneficial [47].

In contrast, however, Ferdinand et al. found in their evaluation of a 7-h CAT program in Victoria that objectives were met if embedded with wider organisational strategies designed to reinforce improvement in Aboriginal employment and health outcomes [48]. It may be argued that the health service has engaged broad strategies and therefore online CAT will suffice in achieving cultural awareness. Yet, to confidently achieve cultural competence, defined as ‘a set of congruent behaviours, attitudes and policies that come together in a system, agency or among professionals to enable that system, agency or those professionals to work effectively in cross-cultural situations’ (p18 [34]), then a more in-depth Cultural Competence Training (CCT) would be beneficial for managers and key staff working alongside Aboriginal employees.

## Conclusion

Eleven strategies have emerged from three themes; safety, equity and pathway, offering mainstream health services insight into how to *mangan dunguludja ngatan* (build strong employment). Cultural safety can be achieved through acknowledging the past and reconciling that through engaging, partnering and collaborating with the Aboriginal community. Visual representations of culture and participation in celebratory activities engender awareness and understanding. The development of local, flexible career development pathways for Aboriginal people facilitates a ‘sense of belonging’ to the health service and a dual ‘sense of pride’ within the community: whereby the Aboriginal person feels proud to represent their community and the community is proud to be represented. Cultural equity is facilitated through mutual learning and reciprocal understanding of difference.

## Data Availability

A de-identified copy of the consultant’s report and field notes are available from the corresponding author.

## Abbreviations

ACCHO: Aboriginal Community Controlled Health Organisation
AEP: Aboriginal Employment Plan
AHA: Allied health assistant
AHLO: Aboriginal Health Liaison Officer
AHMAC: Australian Health Ministers’ Advisory Council
AHW: Aboriginal health worker
AOD: Alcohol and other drugs
CAT: Cultural Awareness Training
CCT: Cultural Competency Training
CHW: Community health worker
CO: Cynthia Opie
DRH: Department of Rural Health
GVH: Goulburn Valley Health
HR: Human Resources
HREC: Human Research Ethics Committee
MoU: Memorandum of Understanding
NAIDOC: National Aborigines and Islander Day Observance Committee
NSW: New South Wales
NT: Northern Territory
PLS: Plain Language Statement
RA: Reconciliation Australia
RAP: Reconciliation Action Plan
RHAN: Rural Health Academic Network
UoM: University of Melbourne
UoS: University of Sydney
